# Prospective evaluation of a dynamic insulin infusion algorithm for non critically-ill diabetic patients: A before-after study

**DOI:** 10.1371/journal.pone.0211425

**Published:** 2019-01-28

**Authors:** Nathanaëlle Montanier, Lise Bernard, Céline Lambert, Bruno Pereira, Françoise Desbiez, Daniel Terral, Armand Abergel, Jérôme Bohatier, Eugenio Rosset, Jeannot Schmidt, Valérie Sautou, Samy Hadjadj, Marie Batisse-Lignier, Igor Tauveron, Salwan Maqdasy, Béatrice Roche

**Affiliations:** 1 CHU Clermont-Ferrand, Service d’endocrinologie, diabétologie et maladies métaboliques, Clermont-Ferrand, France; 2 Pôle Pharmacie, CHU Clermont-Ferrand, Clermont-Ferrand, France; 3 Délégation à la Recherche Clinique et à l'Innovation (DRCI), CHU Clermont-Ferrand, Clermont-Ferrand, France; 4 CHU Clermont-Ferrand, Service de Pédiatrie Générale, Clermont-Ferrand, France; 5 CHU Clermont-Ferrand, Service de Médecine Digestive, Clermont-Ferrand, France; 6 CHU Clermont-Ferrand, Service de Court séjour Gériatrique, Riom, France; 7 CHU Clermont-Ferrand, Service de Chirurgie vasculaire, Clermont-Ferrand, France; 8 CHU Clermont-Ferrand, Pôle Urgences, Clermont-Ferrand, France; 9 CHU Poitiers, Service de Médecine interne, endocrinologie et maladies métaboliques, Poitiers, France; 10 Laboratoire GReD: UMR Université Clermont Auvergne-CNRS 6293, INSERM U1103, Clermont-Ferrand, France; Medical University of Vienna, AUSTRIA

## Abstract

**Introduction:**

Insulin infusion is recommended during management of diabetic patients in critical care units to rapidly achieve glycaemic stability and reduce the mortality. The application of an easy-to-use standardized protocol, compatible with the workload is preferred. Glycaemic target must quickly be reached, therefore static algorithms should be replaced by dynamic ones. The dynamic algorithm seems closer to the physiological situation and appreciates insulin sensitivity. However, the protocol must meet both safety and efficiency requirements. Indeed, apprehension from hypoglycaemia is the main deadlock with the dynamic algorithms, thus their application remains limited. In contrary to the critical care units, to date, no prospective study evaluated a dynamic algorithm of insulin infusion in non-critically ill patients.

**Aim:**

This study primarily aimed to evaluate the efficacy of a dynamic algorithm of intravenous insulin therapy in non-critically-ill patients, and addressed its safety and feasibility in different departments of our university hospital.

**Methods:**

A "before-after" study was conducted in five hospital departments (endocrinology and four “non-expert” units) comparing a dynamic algorithm (during the "after" period-P2) to the static protocol (the “before” period-P1). Static protocol is based on determining insulin infusion according to an instant blood glycaemia (BG) level at a given time. In the dynamic algorithm, insulin infusion rate is determined according to the rate of change of the BG (the previous and actual BG under a specific insulin infusion rate). Additionally, two distinct glycaemic targets were defined according to the patients’ profile: 100–180 mg/dl (5.5–10 mmol/l) for vigorous patients and 140–220 mg/dl (7.8–12.2 mmol/l) for frail ones. Different BG measurements for each patient were collected and recorded in a specific database (e-CRF) in order to analyse the rates of hypo- and hyperglycaemia. A satisfaction survey was also performed. A study approval was obtained from the institutional revision board before starting the study.

**Results:**

Over 8 months, 72 and 66 patients during P1 and P2 were respectively included. The dynamic algorithm was more efficient, with reduced time to control hyperglycaemia (P1 *vs* P2:8.3 *vs* 5.3 hours; HR: 2.02 [1.27; 3.21]; *p*<0.01), increased the number of in-target BG measurements (P1 *vs* P2: 37.0% *vs* 41.8%; *p*<0.05), and reduced the glycaemic variability related to each patient (P1 *vs* P2, %CV: 40.9 *vs* 38.2;*p*<0.05, Index Correlation Class:0.30 *vs* 0.14; *p*<0.05). In patients after the first event of hypoglycemia after having started the infusion, new events were lower (P1 *vs* P2: 19.4 *vs* 11.4; *p*<0.001) thanks to an earlier reaction to hypoglycaemia (8.3% during P1 *vs* 44.3% during P2; *p* = 0.004). With the dynamic algorithm, the percentage of recurrence of mild hypoglycaemia was significantly lower in frail patients (20.5% *vs* 10.2%; *p*<0.001), and in patients managed in the non-expert units (18 *vs* 7.1%, *p*<0.001). The %CV was significantly improved in frail patients (36.9%). Mean BG measurements for each patient/day were 5.5±1.1 during P1 and 6.0±1.6 during P2 (p = 0.6). The threat from hypoglycaemia and the difficulty in using dynamic algorithm are barriers for nurses’ adherence.

**Conclusions:**

This dynamic algorithm for non-critically-ill patients is more efficient and safe than the static protocol, and adapted for frail patients and non-expert units.

## Introduction

Hyperglycaemia is well known to be associated with increased morbidity and mortality in diabetic patients [1–4]. Several studies confirmed the role of earlier glycaemic control in the reduction of the risk of organ failure, systemic infections, and the length of stay in intensive care [3,5–7]. Nevertheless, targeting a strict glycaemic target, *de facto*, exposes the patients to the risk of hypoglycaemia with no much benefits in non-critically ill patients [6,8–10]. First-line intravenous insulin therapy is recommended in intensive care units in order to quickly achieve glycaemic control [11–13]. For this purpose, a wide variety of insulin protocols mainly used in intensive care units have been clinically evaluated [14–17]. To obtain a relatively rapid glycemic control, static algorithms, which determine insulin infusion according instant glycaemia, must be replaced by dynamic ones. In the dynamic algorithms, insulin infusion rate is determined according to the rate of change of the glycaemia, which seems closer to the physiological situation. However, an insulin infusion protocol must meet both safety and efficiency requirements, and the main rules for hypoglycaemia management should be clearly defined [14,18,19]. Indeed, apprehension from hypoglycaemia is the main deadlock with the dynamic algorithms, thus their application remains limited. Consequently, no study prospectively compared a dynamic algorithm to a static protocol in non-critically ill patients. The only three studies already published in non-critically-ill patients were retrospective and observational [20–22]. Passarelli et *al*. performed a retrospective observational study to evaluate a protocol targeting blood glucose (BG) between 140 and 180 mg/dl. A metric evaluation of their data was used. A hypoglycaemic event included all BG values subsequent to the first hypoglycaemic value until BG level was <70 mg/dL. Severe hypoglycaemia was defined as BG as <40 mg/dL. Protocol violation was estimated in 50 patients. Ku et *al*. applied a dynamic protocol for a month in both critical and non-critical care units and compared to the previously managed diabetic patients. Indeed, the percentage of patients who experienced at least one episode of hypoglycemia and marked hypoglycemia were lower with their protocol [20].

Only one “before-after” or “static-dynamic” study has compared the efficacy and safety of a dynamic protocol versus a static one [23]. Again, this study was conducted in an intensive care unit and on a limited number of patients.

Although not well implemented for non-critically ill patients, insulin infusion is often used to manage acute situations such as ketoacidosis, hyperosmolar coma, and during steroid therapy, artificial nutrition, and fasting periods [24,25].

Within our University Hospital Centre of Clermont-Ferrand, 29 static different insulin infusion protocols have been identified in different departments of non-critically ill units. Thus, harmonization was essential to improve the quality of diabetes management in the institution. For this purpose, we preferred to develop a dynamic algorithm. In this perspective, we developed a dynamic algorithm for insulin infusion in harmony with the recommendations, and the human and logistical constraints of the healthcare teams in units managing non-critically ill patients. The elaboration process of this protocol was previously published [26].

This study aimed to assess the efficacy and safety of a dynamic standardized algorithm for insulin infusion, and to evaluate its feasibility before implementation on the whole hospital structure. In order to assess the algorithm, we have implemented a before-after study through which we compared the dynamic algorithm (after period) to a static algorithm used in in the expert unit (endocrinology) and in four non-expert units that represent of the diversity of the medical and surgical wards.

## Materials and methods

A “before-after” study was performed on diabetic non-critically ill patients within five different medical care units of the university hospital of Clermont-Ferrand (departments of endocrinology or “the expert unit”, and four “non-expert units”, namely: digestive medicine, post-emergency, geriatrics, and vascular surgery). The classical static protocol used in such units during the “Before” period (P1) was evaluated over 4 months (from 2015, October to 2016, January) (**S1 Fig**). The static protocol refers to a fixed insulin infusion rate taking in consideration only blood glucose level at a given time, with the infusion rate changed by a fixed increment for all patients. It does not take in consideration the evolution of BG under the previous insulin infusion rate (insulin sensitivity during a period of time).

During the “After” period (P2), the static protocol was retrieved and the new dynamic algorithm was implemented in all the departments and applied for 4 months (from 2016, July to 2016, October). A dynamic algorithm refers to a specific rhythm of adaptation of insulin infusion rate that takes in consideration the rate of change in BG (the difference between the previous and the present BG) and insulin infusion rate. Thus, it takes in consideration the body sensitivity to a given insulin rate to adapt the next rate. It ideally discovers the impact of insulin resistance and carbohydrate exposure during the course of treatment, such that the rate of insulin delivery may be revised when those variables change over time. Our dynamic algorithm defines the target for BG, the rules for initial insulin infusion rate assignment, the rules for management and prevention of hypoglycemia, and the rules to include subcutaneous injections before meals. Both the current glucose measurement and also the rate of change of glucose are among the minimal inputs necessary for periodic revision of the insulin infusion rate.

Before the implementation of the new algorithm, several training sessions for the nurses and physicians were set up in order to present its main specificities and modalities. Two distinct glycaemic targets were defined according to the patients’ profile: for patients with a generally good or vigorous status, the glycaemic target was comprised between 100–180 mg/dl (5.5–10 mmol/l); for patients with multiples comorbidities (heart and/or liver failure, malnutrition, chronic renal failure, cognitive troubles, history of repeated severe hypoglycemia) and/or unstable diabetic retinopathy or ischemic heart disease (frail patients), the fixed target was larger, between 140–220 mg/dl (7.8–12.2 mmol/l) [10,27–30]. High HbA1c (previous hyperglycemia) was not included as a criteria of frailty as reported by some authors [1,31,32].

The protocol included detailed guidelines to correct hypoglycaemia with different thresholds according to its intensity and patient’s profile. For “frail patients”, BG between 70–100 mg/dl and <70mg/dl (<3.9 mmol/l) were respectively considered as mild and marked hypoglycaemia. For the vigorous ones, BG between 50–70 mg/dl (2.8–3.9 mmol/l) and <50mg/dl (<2.8 mmol/l) were respectively considered to define mild and marked hypoglycaemia (**S2, S3 and S4 Figs**).

Inclusion criteria were patients with diabetes over 18 years old and requiring intravenous insulin infusion. Exclusion criteria were patients without health insurance, deprived judicial or administrative liberty, or unable to express their non-opposition to the protocol.

The Commission of Drug and Medical Devices (COMEDIMS) of the University Hospital of Clermont-Ferrand validated the protocol. Before starting the P1 period, a study approval was obtained from the institutional revision board. The Committee for the Protection of Patients (CPP) authorized this study (2015 / CE 68). Patients of both periods were prospectively included, and the non-opposition to the use of the medical data was obtained from all participating patients during P1 and P2. This study was declared to the national commission of informatics and freedom under the number 0137.

At inclusion, demographic and clinical data were recorded for each patient and collected within an e-CRF (electronic Case Report Form). In addition, all diabetes-related data were collected: type and medical history of diabetes, its complications and treatment. The indication of the intravenous insulin infusion or its discontinuation were also reported. Capillary BG measurements and several other data were recorded in the e-CRF, several times daily. These data include infusion rate, quantity of glucose administered in case of hypoglycaemia, and the algorithm’s compliance (applied infusion rate, and reaction to hypoglycaemia or hyperglycaemia). BG measurements were performed every 2 hours (including controls before and after each meal) until the BG target was reached, and every 4 hours during the following time. BG was only determined by capillary glycaemia measurement without simultaneous laboratory measurements.

The rate of BG change reflects the rate of increment or reduction of BG levels in a patient. The percentage of BG measurements within the target refers to the percentage of all measurements that was within the fixed target for a patient. The percentage of hypoglycemia per patient refers to the average of hypoglycemia episodes for each patient, then averaged for all the cohort.

The primary objective of the study consisted in evaluating the efficacy of the dynamic algorithm compared to the previous static one. Algorithm’s efficacy was estimated thanks to the time lapse needed to reach the pre-defined glucose range, the percentage of BG measurements within the target, and the glycaemic variability (including coefficient of variation for glucose or %CV calculated according to Monnier et *al*.[33]) after reaching the target. The secondary objective was to evaluate the safety and the feasibility of the dynamic algorithm. Safety evaluation was based on the rate of hypoglycaemia (mild or marked as defined above), and hyperglycaemia above 250 mg/dl (13.9 mmol/l) once targets have been reached. Indeed, we considered that when BG was under 250 mg/dl (13.9 mmol/l), the major hyperglycemia-related complications (DKA, hyperosmolar coma) are absent. Under this cutoff, the glycemic variability remained acceptable [6,19,20].

Feasibility was estimated by considering the compliance and satisfaction of the medical team. Nursing compliance was estimated by recording the number of protocol violations by the nurse. The latter was defined as the number of times that the nurse interfered on the total blood glucose measurements. Moreover, at each rate assignment, we reported any non-adjusted insulin infusion or modified the monitoring frequency (above 20 minutes) beyond what was pre-defined by the algorithm. Nursing workload was indirectly measured by the frequency of blood glucose measurements performed. Satisfaction of nurses and physicians was assessed using a satisfaction survey given at the end of the clinical study, according the following scale, 0: not satisfied; 10: fully satisfied.

### Statistical analysis

We calculated that a sample of 50 patients per period was necessary and relevant to compare the rate of hypoglycemia between static (P1) and dynamic (P2) periods. Indeed, considering a minimum of 5 to 6 BG measurements per day per patient and a follow-up of 7 days, we supposed that 1500 measures by period would be compared. Despite the lack of information concerning within patient variability, we could suppose that such sample size allows to highlight an absolute difference around 5% for a rate of hypoglycemia at 10% in static period, with a two-sided type I error at 5% and a statistical power greater than 90%.

Statistical analysis was performed using Stata software (version 12, StataCorp, College Station, US). All tests were two-sided, with α = 0.05. Continuous data were expressed as mean ± standard deviation or as median [interquartile range], according to statistical distribution. Categorical parameters were presented as frequencies and associated percentages.

When statistical unit was the patient, continuous data were compared between groups (especially static period versus dynamic period) by Student t-test or Mann-Whitney test when assumptions of t-test were not met. The normality was studied with Shapiro-Wilk test and the homoscedasticity with Fisher-Snedecor test. Concerning categorical data, Chi-squared or Fisher’s exact tests were performed. The rates of hypoglycemia and hyperglycemia per patient were compared between groups by zero-inflated negative binomial models. The time to reach the target, considered as a censored data, was estimated by the Kaplan-Meier method and difference between the two periods was assessed by Cox regression. The proportional-hazard hypothesis was studied using Schoenfeld’s test and plotting residuals. Results were expressed as hazard ratios and 95% confidence interval.

When statistical unit was the BG measurements, statistical analysis was conducted using mixed models to take into account the repeated measurements per patient (patient as random effect to measure within and between subject variability): linear mixed models (LMM) for quantitative variables and generalized linear mixed models (GLMM) with logit link function for binary parameters. Residuals normality of these models was studied as described previously. Then, multivariate analyses were performed to adjust on possible confounding parameters determined according to univariate results and to clinical relevance: age, oral feeding or not, known or discovered diabetes. Finally, glycemic variability has been evaluated with intraclass correlation coefficients (ICC) and coefficients of variation (CV). ICC is a reliable statistical description of the quantitative measurements or units between groups describing how strongly units in the same group resemble each other. It helps to assess of consistency or reproducibility of BG collected thanks to different measurements or different timing.

## Results

### General characteristics

From October, 2015 to October, 2016, 138 patients were included, 72 patients during the “before” or static period (P1), and 66 patients during the “after” or dynamic period (P2), with a slight predominance of frail patients during both periods (59.7% and 56.1% of patients respectively). Main patients’ characteristics are summarized in **Table 1**. Both cohorts were well matched regarding the demographical data and the characteristic related to diabetes. Most patients had type 2 diabetes that had been diagnosed for 10 years (84.7% during P1 and 81.5% during P2), and poorly controlled: mean HbA1c was 10.2±2.8% (88 mmol/mol) during P1 and 10.8±2.8% (95 mmol/mol) during P2. Diabetes complications were present in about half of the cases. The main reason for insulin infusion initiation was significant hyperglycaemia with oral antidiabetic drugs or subcutaneous insulin injections. Moreover, it was initiated because of ketoacidosis, hyperosmolar coma, and during peri-surgical period. Insulin infusion was stopped after achieving the glycaemic control in both periods (P1 *vs* P2: 83% *vs* 88%) before transition to subcutaneous insulin or oral drugs. The median intravenous insulin infusion duration did not significantly vary.

**Table 1:** Population characteristics during «before» and «after» periods (P1 *vs* P2). Data are mostly expressed as number of patients (percentage of patients)±Standard Deviation. NPO: Nil Per Os; PN: Parenteral Nutrition; EN: Enteral Nutrition. Univariate analysis was adjusted by a multivariate one.

	STATIC (P1)	DYNAMIC (P2)	*p*
**Number of patients**	72	66	
**Female (%)**	26 (36.1)	22 (33.3)	0.73
**Age (years)**	65.8 ± 17.4	62.8 ± 18.3	0.32
**BMI (kg/m**^**2**^**)**	29.0 ± 7.6	28.5 ± 9.8	0.73
**Types of diabetes: number (%)**			
Type 1	7 (9.7)	7 (10.8)	
Type 2	61 (84.7)	53 (81.5)	0.89
Other	4 (5.6)	5 (7.7)	
**Duration of diabetes (years)**	10.8 [0.3; 20.0]	8.7 [0.1; 16.6]	0.29
**Complications of diabetes: number (%)**	43 (59.7)	30 (45.5)	0.89
**Diabetes treatment: number (%)**			
None	8 (11.1)	17 (25.7)	0.30
Diet	4 (5.5)	2 (3.0)	
Oral antidiabetics	21 (27.8)	18 (27.3)	
Insulin alone	21 (29.2)	20 (30.3)	
Insulin+oral antidiabetics	18 (25.0)	9 (13.7)	
**Diabetes discovery: number (%)**	6/72 (8.3)	12/66 (18.1)	0.09
**HbA1c at inclusion (%)**	10.2±2.8	10.8±2.8	0.24
**Glycaemia at inclusion (mg/dl)** **(mmol/l)**	247±0.99 13.7±5.5	258±113 14.3±6.3	0.55
**Frail patients: number (%)**	43 (59.7)	37 (56.1)	0.58
**Endocrinology unit: number (%)**	36 (50)	38 (57.6)	0.16
**Non-expert units: number (%)**			
Vascular surgery	12 (16.7)	3 (4.5)	
Digestive medicine	12 (16.7)	9 (13.6)	
Post emergency	7 (9.7)	11 (16.7)	
Geriatric	5 (6.9)	5 (7.6)	
Other (NPO, PN, EN)	9 (12.5%)	16 (10.6%)	**0.04**
**Number of BG measurements**			
Overall	5.55±1.1	6.01±1.65	0.06
Vigorous patients	5.68±1.18	5.4±1.56	0.44
Frail patients	5.45±1.14	6.49±1.11.59	**0.002**

Overall, 1517 and 1324 BG measurements were performed during P1 and P2 respectively. Indeed, 5.5±1.1 BG measurements for each patient/day were performed during P1 and 6.0±1.6 during P2 **(Table 1, S1 Table)**). Missing data represented only 1.2% of the overall BG measurements (19/1536) during P1 and 2.1% during P2 (29/1353).

### Primary end point: Efficacy of the dynamic algorithm

**Fig 1:**
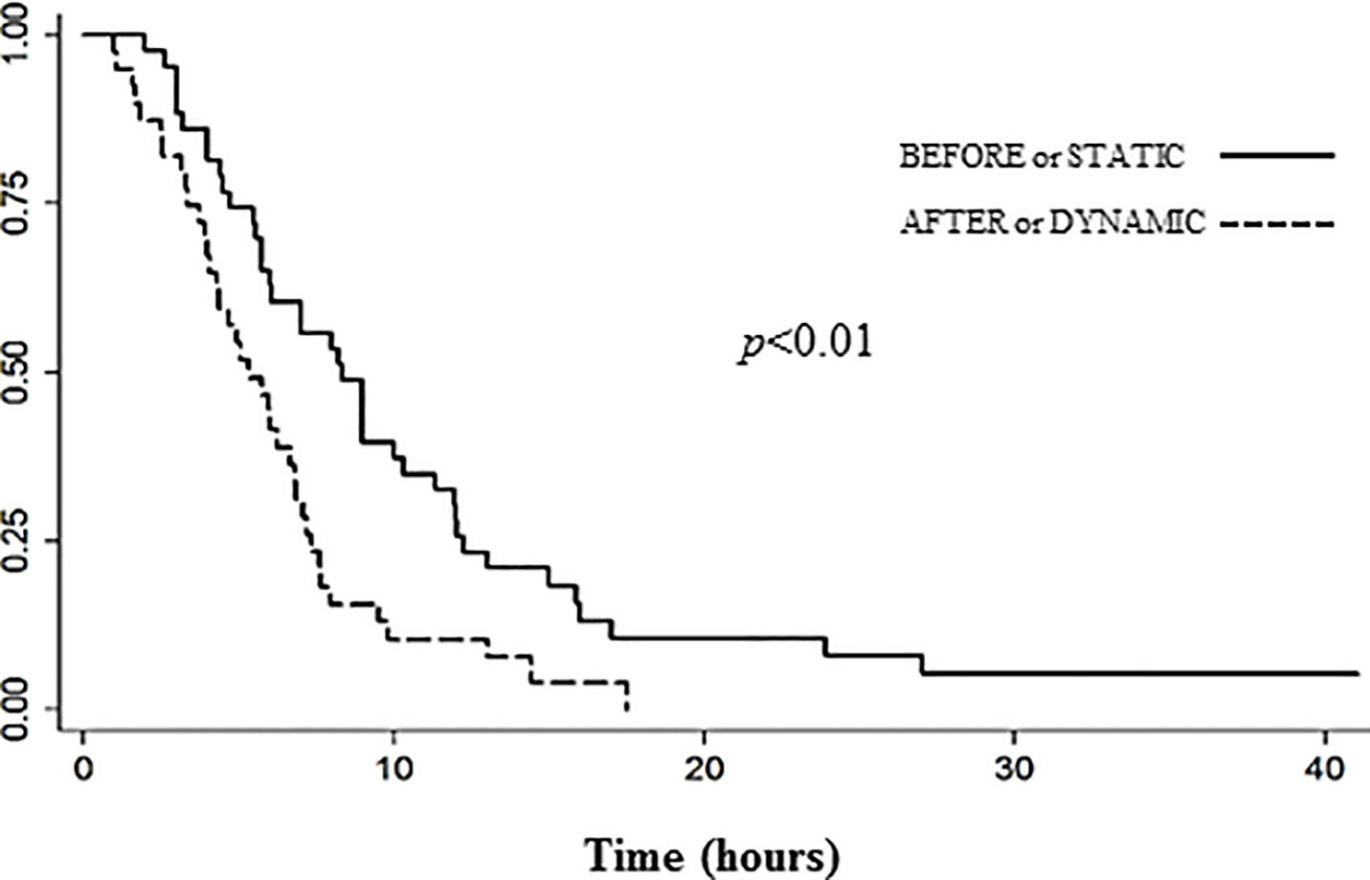
Time to reach the glycemic target demonstrating the efficacy of a dynamic algorithm. Fifty percent of patients achieved the glycemic target in 8.3 hours [4.7; 12.2] during P1 «static protocol» *vs* in 5.3 hours [3.4; 7.3] during P2 «dynamic algorithm».

To study the efficacy of the dynamic algorithm, 3 parameters were considered: the percentage of BG measurements within the pre-defined target, the mean time needed to reach the target, and the glycaemic variability. With the dynamic algorithm, the % of BG measurements within the target was more important compared to the previous static protocol (P1 *vs* P2: 37% *vs* 41.8%; *p*<0.05). The target was more quickly reached during the “after” period (P1 *vs* P2: 8.3 hours [CI: 3.4; 7.3] *vs* 5.3 hours [CI: 4.7; 12.2]; *p*<0.01) (**Fig 1**). The dynamic protocol was able to significantly reduce the glycaemic variability related to each patient (Index Correlation Class for P1 *vs* P2: 0.30 *vs* 0.14; *p*<0.05), especially in frail ones (interclass correlation coefficient: 0.32 [0.22; 0.44] *vs* 0.12; [0.06; 0.21] (*p*<0.05). The coefficient of variation of glycaemia consequently reduced from 40.9 to 38.2 (36.9 in frail patients) (**Table 2, S2 Table)**.

**Table 2:** Efficacy of the dynamic algorithm evaluated by the global glycemic variation expressed by Index Correlation Class (ICC) and the percentage of coefficient variability for glucose (%CV).

	Global glycemic variation			%CV once BG in the target	
	STATIC ICC [IC 95%]	DYNAMIC ICC [IC 95%]	*p*	STATIC	DYNAMIC
**Total**	0.30 [0.23 ; 0.39]	0.14 [0.09 ; 0.22]	**<0.05**	40.9	38.2
**Vigorous**	0.27 [0.16 ; 0.41]	0.16 [0.07 ; 0.31]	0.39	41.7	39.8
**Frail**	0.32 [0.22 ; 0.44]	0.12 [0.06 ; 0.21]	**<0.05**	40.1	36.9
**Endocrinology**	0.31 [0.20 ; 0.45]	0.11 [0.05 ; 0.21]	**<0.05**	39.1	38.5
**Non-expert**	0.30 [0.20 ; 0.43]	0.15 [0.07 ; 0.28]	0.10	41.6	37.1

### Secondary end points: Safety and feasibility of the dynamic algorithm

To evaluate the safety of the dynamic algorithm, the main parameters taken in consideration were hypo- and hyperglycaemia. Compared to the static protocol, the proportion of patients who experienced at least one hypoglycaemia did not significantly differ with the dynamic algorithm (40.3% during P1 *vs* 48.5% during P2, *p* = 0.33). Hypoglycaemia rate in the cohort (percentage of hypoglycaemia over all BG measurements) was overall similar (P1 *vs* P2: 7.3% *vs* 4.9%; *p* = 0.80). But, among those who had the first event of hypoglycemia after having started the infusion, the percentage of subsequent hypoglycaemia per patient was lower with the dynamic algorithm (P1 *vs* P2: 19.4% *vs* 11.4%; *p*<0.001) (**Table 3**). Besides, the rate of hypoglycaemia was 7 times higher in the ‘frail’ population with the static protocol (frail *vs* vigorous: 10.9% *vs* 1.4%; *p*<0.001). With the dynamic algorithm, the rate of hypoglycaemia tended to be lower in this population (P1 *vs* P2: 10.9% *vs* 5.2%; *p* = 0.06), and the percentage of recurrence of mild hypoglycaemia in a patient after his first episode was significantly lower in frail patients treated with the dynamic algorithm compared to the static one (frail P1 *vs* frail P2: 20.5±14.3% *vs* 10.2±7.7%; *p*<0.001) (**Table 4**).

**Table 3:** Evaluation of the safety of the dynamic algorithm « after period or P2 » *vs* the static protocol « before period or P1». Mild hypoglycemia is defined by a BG level <70 mg/dl (<3.9 mmol/l) in vigorous patients and <100 mg/dl (5.5 mmol/l) frail patients. Marked hypoglycemia is defined by a BG level <50 mg/dl (<2.8 mmol/l) in vigorous patients and <70 mg/dl (<3.9 mmol/l) frail patients. Hyperglycemia after achieving the target is defined by BG>250 mg/dl (>13.9 mmol/l).

	STATIC 72 patients	DYNAMIC 66 patients	*P*
**Mild hypoglycemia**			
Number of blood glucose measurements	1517	1324	0.8
At least one hypoglycemia [n (%)]	29 (40.3)	32 (48.5)	0.31
Hypoglycaemia episodes [n (%)]	110 (7.3)	65 (4.9)	0.8
% of hypoglycemia per patient (mean %±SD)	19.4±13.9	11.4±7.1	**0.001**
**Marked hypoglycemia**			
At least one marked hypoglycemia [n (%)]	10 (13.9)	10 (15.2)	0.83
% of episodes per patient (mean %±SD)	10.1±6.2	8.7±5.1	0.57
Marked hypoglycaemia episodes [n (%)]	23 (1.5)	15 (1.1)	0.97
**Hyperglycaemia**			
At least one hyperglycemia after reaching the target [n (%)]	47 (68.1)	41 (65.1)	0.71
% of hyperglycemia episodes per patient [mean±SD]	23.4±16.3	21.3±13.0	0.83
Hyperglycemia [n/overall BG measurements]	228/1350	214/1211	0.94

**Table 4:** Evaluation of the safety of the dynamic algorithm *vs* the static protocol according to patients’ profile (vigorous *vs* frail patients). *p1* represents the statistical analysis between frail and vigorous patients treated with the static protocol; *p2* represents the statistical analysis between frail and vigorous patients treated with the dynamic algorithm; *p3* represents the statistical analysis between frail patients treated with the static protocol and the dynamic algorithm; *p4* represents the statistical analysis between vigorous patients treated with the static protocol and the dynamic algorithm. Mild hypoglycemia is defined by a BG level <70 mg/dl (<3.9 mmol/l) in vigorous patients and <100 mg/dl (<5.5 mmol/l) frail patients. Marked hypoglycemia is defined by a BG level <50 mg/dl (<2.8 mmol/l) in vigorous patients and <70 mg/dl (<3.9 mmol/l) frail patients. Hyperglycemia after achieving the target is defined by BG>250 mg/dl (>13.9 mmol/l). (…) represents the % value over all BG measurements.

	STATIC			DYNAMIC				
	Frail	Vigorous	*p1*	Frail	Vigorous	*p2*	*p3*	*p4*
**Mild hypoglycaemia**								
Mild hypoglycaemia rate	**102/934 (10.9)**	**8/583 (1.4)**	**<0.001**	45/870 (5.2)	20/454 (4.4)	0.51	**0.06**	0.07
% of mild hypoglycaemia per patient after the first hypoglycemia	**20.5±14**	12.8±10	0.21	**10.2±7**	13.9±5	0.15	**<0.001**	0.77
**Marked hypoglycaemia**								
Marked hypoglycaemia rate	20/934 (2.1)	3/583 (0.5)	0.17	12/870 (1.4)	3/454 (0.7)	0.36	0.87	0.87
% of marked hypoglycaemia per patient after the first hypoglycaemia	9.6±6	12.2±8	0.59	9.2±5	7.7±3	0.65	0.89	0.25
**Hyperglycaemia**								
Hyperglycaemia after reaching the target	136/849 (16.0)	92/501(18.4)	0.81	161/809 (19.9)	53/402 (13.2)	0.25	0.60	0.37
% of hyperglycaemia per patient after reaching the target	25.0±18	21.0±13	0.40	21.9±12	20±13	0.72	0.50	0.90

The percentage of hypoglycaemia was largely reduced in the “non-expert units” during P2 (P1 *vs* P2: 18±15% *vs* 7.1±4.9%, *p*<0.001), and to a lesser extent in the endocrinology department. This reduction was particularly significant in the frail patients population (P1 *vs* P2: 20.5±14.3% *vs* 10.2±7.7%; *p*<0.001).

The percentage of marked hypoglycaemia was extremely rare accounting for <5% of BG during P1 and P2 (1.4%±4.2 during P1 and 1.3%±3.7 during P2) (**Table 3**).

Hyperglycaemia after achieving the target was reported in about 17% of patients in both periods (**Table 3**). Hyperglycaemia rates were similar whatever the used protocol, unit of management or type of patient. No case of acute complications such as ketoacidosis or hyperosmolar coma was reported during the dynamic algorithm period.

To evaluate the feasibility of the dynamic algorithm, compliance and satisfaction of the medical team were taken in consideration. The adherence to the dynamic algorithm was as satisfactory as that under the static protocol (used for many years and well implemented by the teams), or even better. Under the dynamic protocol, the deviation from BG monitoring schedule was only observed in 32% of the presumed BG measurements, while it was observed in 57% of cases under the static protocol (p<0.001) (**Table 5**). Deviation from the theoretical insulin infusion rate concerned 1/5 patients in both periods (20.6% *vs* 21.7%). Pre-prandial insulin adjustment was well conducted in 65.4% of cases with the dynamic protocol. Deviation from the algorithm led to hypoglycaemia in 60% of the cases during P1 and P2. Overall, 40% of marked hypoglycaemia were preceded by violation of the presumed insulin rate during P2 compared to 8% during P1. Hypoglycaemia was best managed during P2 (P1 *vs* P2: 8% *vs* 44% of hypoglycaemia; *p* = 0.004); this issue also concerned marked hypoglycaemia (P1 *vs* P2: 32% *vs* 87%; *p* = 0.09) **(Table 5).**

**Table 5:** Feasibility and compliance to the algorithms. Comparison between « before or static » and « after or dynamic» periods.

PARAMETER	STATIC	DYNAMIC	
Deviation from monitoring rate [n/n (%)]	867/1517 (57.2)	423/1324 (31.9)	**<0.001**
Deviation from insulin infusion rate [n/n (%)]	312/1517 (20.6)	287/1323 (21.7)	0.73
Pre-meal adjustement [n/n (%)]	197/401 (49.1)	204/312 (65.4)	0.28
Mild Hypoglycaemia management [n/n (%)]	9/109 (8.3)	27/61 (44.3)	**0.004**
Marked hypoglycaemia management [n/n (%)]	7/22 (31.8)	13/15 (86.7)	**0.09**

Satisfaction of both physicians and nurses was assessed at the end of the study (according the following scale: 0 means not satisfied; and 10 means fully satisfied). The response rate was 45% and 100% for nurses and physicians respectively. Among respondents, 56.8% of nurses and 64% of physicians attended the training session, which satisfied more than 85% of them. Concerning the dynamic algorithm itself, the nurses were generally not satisfied (satisfaction mark: 3.4**±**2.3/10). Conversely, the physicians were generally satisfied (7.2±1.6/10). The difficulties of feasibility constituted the main barrier, especially the complexity of reading and the time required for dose adjustment. Moreover, glycaemic monitoring frequencies were judged excessive by nurses, attributing a rate of 2.8/10 for this item, though, daily BG measurements did not significantly vary between P1 and P2, except the third day of inclusion, and in frail patients with more BG measurements (5.9±2.0 during P1 vs 7.7±2.1 during P2; p<0.001) (**Table 1, S1 Table**).

**Fig 2:**
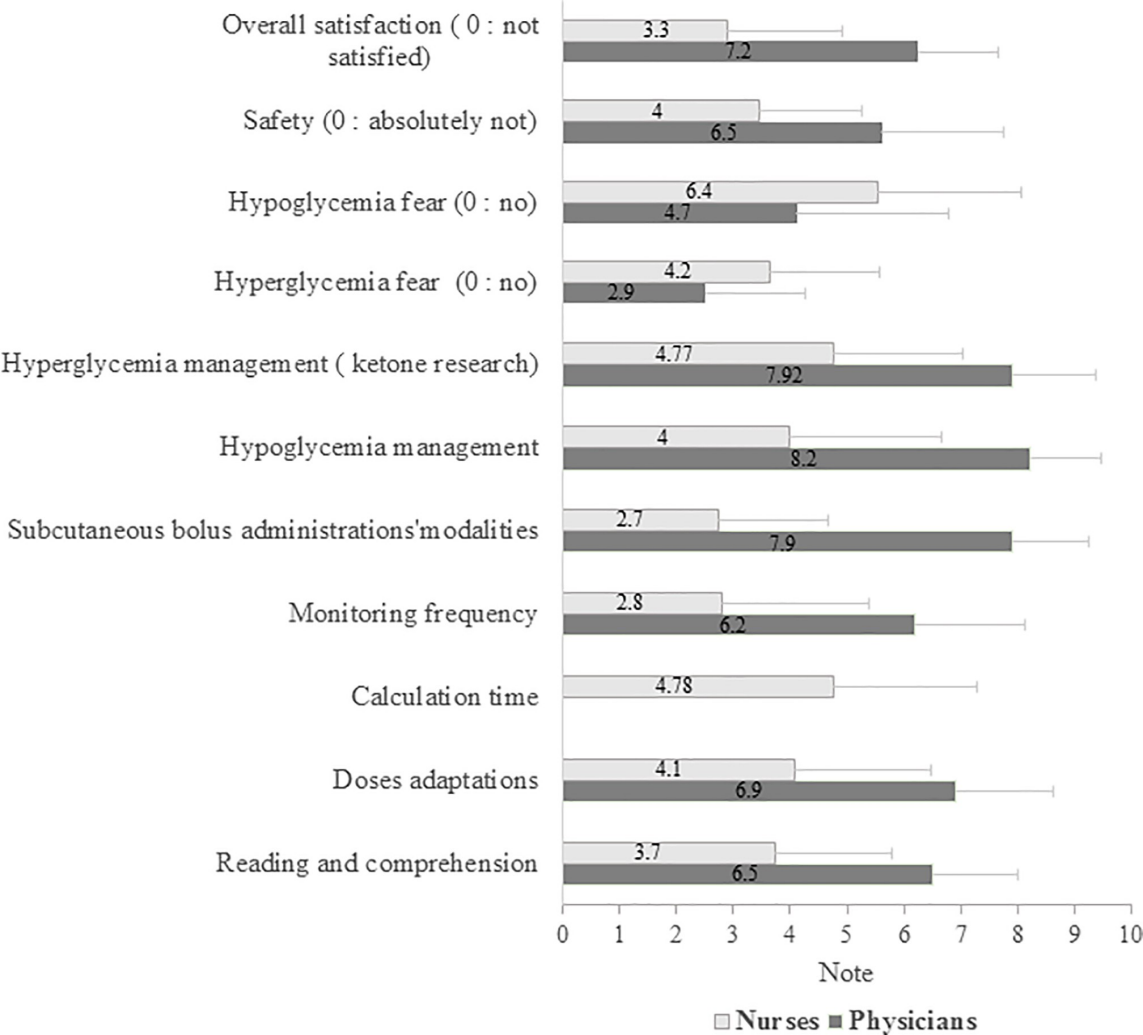
Evaluation of satisfaction and apprehension from the dynamic algorithm of the medical staff. Scales of satisfaction: 0 means not satisfied; 10 means totally satisfied. Scales of apprehension: 0 means no fear from an undesirable event; 10: means maximal apprehension. Notes of evaluation are presented by mean with its standard evaluation.

Nurses considered the dynamic algorithm not safe enough with a significant personal fear from hypoglycaemia [6.4±2.9/10 on a scale of 0 (no fear) to 10 (great fear)]. This feeling was less important for physicians who attributed a score of 4.7±3.1/10). By contrast, fear from hyperglycaemia was less important (4.2±2.25/10 and 2.9±2.0/10 for nurses and physicians respectively). Paradoxally, the satisfaction survey was mainly answered by nurses with low rate of training on diabetes, since only one nurse out of ten was previously trained on diabetes during the last three years (**Fig 2**).

## Discussion

This study demonstrates the efficacy, safety and feasibility of a dynamic algorithm compared to the previous static one in units managing non-critically ill patients. For the first time, this study has prospectively compared a dynamic algorithm to a static protocol in non-critically ill patients since all previous studies were retrospective and observational [20–22]. Ku et *al*. applied a dynamic protocol for a month in both critical and non-critical care units and compared to the previously managed diabetic patients. Indeed, the percentage of patients who experienced at least one episode of hypoglycemia and marked hypoglycemia were lower with their protocol [20]. However, in this study, the prospective application of a dynamic algorithm was compared to the previous preexistent BG (retrospective) with no details on the insulin infusion protcols and their heterogeneity. Only one before-after study has compared the efficacy and safety of a dynamic protocol, but it was conducted in an intensive care unit [23]. The clinical validity of our algorithm has been assessed on a relatively large group of patients, well representative of the diversity of diabetes (diabetes unit, surgical or digestive diseases, and vigorous as well as frail patients).

With the dynamic algorithm, we have shown an improvement of hypoglycaemia rates in the units with no specific experience on diabetes management, especially in frail patients.

The dynamic algorithm enables a faster and more efficient achievement of the glycaemic target represented by the time needed to be in-target and the percentage of BG measurements within the target respectively. Furthermore, the rate of BG change and the coefficient of variation for glucose (%CV) were lower with the dynamic algorithm. As suggested by Monnier et *al*., lower %CV defines a stable glycaemia and is associated with a reduced risk of hypoglycaemia [33]. The cut-off is fixed to 36% (obtained in patients with no hypoglycaemic agents) to define stable and unstable glycaemia. Indeed, thanks to the dynamic algorithm, %CV was reduced especially in frail patients, where it was near 36%. Unfortunately, despite this positive result on the efficacy, no difference in total insulin infusion duration was observed.

The definition of two distinct profiles according to the degree of patient’s illness led to reduce the global BG variability in patients treated with the dynamic algorithm. However, the individual glycaemic variability of each patient remained identical to that in static protocol. Nevertheless, managing to reduce variability is an important challenge, as we know the negative predictive effect of glycaemic variability on mortality rates [34]. Clergeau et *al*. demonstrated the superiority of a dynamic protocol compared to a static one on variability among critically-ill patients [35]. Choosing larger glycaemic targets in our study to reduce hypoglycaemia participates, perhaps, in the failure of reduction of the individual variability.

Despite the same frequency of the first event of hypoglycaemia between both periods, subsequent hypoglycaemic rates after the first event were lower with the dynamic algorithm, especially in more vulnerable patients and in the non-expert units. The latter two points are crucial due to the elevated hypoglycaemia-related mortality in the frail patients, and the elevated risk of hypoglycaemia in the non-expert units. On one hand, these results are explained by a better reaction to hypoglycaemia, which was more often respected and applied earlier than in the “before” period. On the other hand, a larger glycaemic target adjusted according to patient’s profile, contributed to reduce the risk of hypoglycaemia in such patients. These results enhance the benefits of the distinct targets according the frailty or previous dysglycemia, as suggested by several studies [31,32]. Indeed, Krinsley et *al*. in a 2 years interventional trial demonstrated a significant reduction in mortality by using a loose protocol with larger targets (110–160 mg/l *vs* 80–140 mg/l) for patients with poorly controlled diabetes mellitus (HbA1c above 7%) [32]. Moreover, Egi et *al*. demonstrated an association between a chronic pre-morbid hyperglycaemia and the acute hypoglycaemia risk [31]. In the literature, hypoglycaemia rates are similar or lower than data reported by three other studies performed on non-critically-ill populations. Indeed, in our study, 18.2% of patients have experienced at least a hypoglycaemic episode below 60 mg/dl (<3.33 mmol/l), with a low hypoglycaemia incidence of 1.1%. In comparison, hypoglycaemia was reported in 22–32% of inpatients [20–22], while hypoglycaemia (<60mg/dl or <3.33 mmol/l) incidence reported on BG measurements was estimated between 0.1% and 1.5% in the literature [36–38]. Moreover, the proportion of marked hypoglycaemia below 50 mg/dl (<2.77 mmol/l) and 40 mg/dl (<2.22 mmol/l) remains rare with our dynamic algorithm (respectively, 0.4% and 0.2% of BG measurements under the dynamic protocol).

After achieving the target, hyperglycaemia represents about 17.7% of the BG measurements under the dynamic algorithm, in the same proportion as in the study of Passarelli et *al* [21], while the rates reported in another study ranged from 35% to 48% during the 2^nd^ and 3^rd^ days of insulin infusion [20]. Indeed, hyperglycaemia is arbitrary and not following hypoglycaemia, suggesting that sugar substitution was warranted. High rates of hyperglycaemia in our dynamic algorithm could be overestimated due to a higher number of patients with enteral nutrition included during P2.

Several studies have assessed nurses’ adherence after implementation of a new protocol, and reported rates vary from 55% to 88%, according to the simplicity, computerization of the protocol, and the confidence of the teams in the safety and efficacy of the protocol [39]. Our satisfaction survey reveals a low adherence by nurses while in contrast, most physicians were satisfied. Lack of feasibility in a non-critically ill unit seems to be one of the major barriers to adherence. The complexity of the dynamic algorithm needs calculation and more time to read and to understand, which is not always compatible with the workload in the medical or surgical units. Computerization might improve both the feasibility and the adherence to the protocol as suggested by Rood et *al*., who have shown a better compliance to monitoring and infusion adjustment thanks to a computerized version of a protocol [40]. In our study, even if the deviation from the protocol was not higher in the dynamic algorithm than previously, most hypoglycaemic episodes and about a half of hyperglycaemia cases were preceded by the lack of respect of the algorithm. These data suggest that hypoglycaemia and hyperglycaemia rates could be lower if the protocol is well respected, suggesting the necessity to improve the adherence to obtain a prompt glycaemic control.

The second barrier to adherence is the resistance to change an old well-implemented protocol that has been used for the last 20 years *versus* a new one implemented for a few months. The fear associated to this dynamic protocol was as important as it was not often used by each individual nurse, since about 25% of the nurses used the dynamic protocol less than three times. This frequency of use is lower than the levels reported by Malesker et *al*.; in their study, 39% of nurses used the protocol more than 20 times [41].

This study underlines the need of nurses’ training on diabetes in order to improve their adhesion. In fact, only 1 out of 10 nurses had received training on diabetes during the three previous years, which could explain the low adherence to the management protocols for hypoglycaemia or hyperglycaemia. The regular support set up during the dynamic protocol implementation for the “after” period was not sufficient. A more intensive coaching by an expert team in the management of diabetes is required to create awareness within the teams to the issue of glycaemic control in patients with diabetes on one hand, and to resolve the team’s difficulties met during daily practice in order to improve their adherence, on the other hand.

Nevertheless, the implementation of this dynamic algorithm had a positive effect, since it upheld the awareness to the quality required for an intravenous insulin infusion used in medical or surgical departments. Even if occasionally this treatment generates fear and apprehension, this dynamic protocol has created awareness within the teams to the need of narrow glycaemic monitoring, and the observance of the specific instructions for the management of hypoglycaemia or hyperglycaemia.

The major drawback of the study is the design with a sequential non randomized model. Furthermore, the frequency of BG measurements could vary according to the evolution of BG and insulin rate adjustment. The non-pre-specified frequency might induce a measurement bias, by non-reporting true hyperglycaemia or hypoglycaemia. However, reactive BG measurements after a great decrease in glycaemia or significant modification of insulin rate could often contribute to the increased monitoring frequency. Indeed, the number of BG measurements did not significantly vary between both protocols. Furthermore, this study demonstrates the efficacy and safety of a dynamic algorithm in non-critical units. Despite the complexity of such studies and the limited relevant outcomes in a short time, the benefits on long term diabetes control and mortality would be interesting.

## Conclusions

This prospective comparative clinical study proved the efficacy and safety of a dynamic insulin infusion algorithm in non-critically ill patients. Glycaemic target was achieved faster and more frequently, without increasing the risk of hypoglycaemia; hypoglycaemia rates reduced in the units with no specific experience on diabetes management, and in frail patients thanks to a better reaction to hypoglycaemia. Glycaemic variability significantly decreased thanks to the definition of distinct glycaemic targets adapted to patient’s profile. In order to harmonize this protocol within the hospital, some adjustments are required including its computerization to improve the feasibility of the protocol. Regular coaching by expert teams during the first steps of implementation of this protocol is necessary in order to improve nurses’ adherence.

## Supporting information

S1 Table Mean number of blood glucose (BG) measurement per day: Comparison between « before or static » and « after or dynamic» periods. For each day of insulin infusion, the mean number of BG measurements/patient with standard deviation are given.
(DOCX)

S2 Table Evolution of mean blood glucose (BG) levels during both before and after periods. For each timing of insulin infusion, the mean BG (mg/dl) with the standard deviation are given.
(DOCX)

S1 Fig Static protocol used during the before period. (PDF)

S2 Fig FrontPage of the dynamic algorithm for insulin infusion adapted for vigorous patients. (PDF)

S3 Fig FrontPage of the dynamic algorithm for insulin infusion adapted for frail patients. (PDF)

S4 Fig Back page of the algorithm with specific protocol recommendations. (PDF)
