# Supplementary material for: Prospective evaluation of a dynamic insulin infusion algorithm for non critically-ill diabetic patients: A before-after study
Source: PLoS One. 2019 Jan 28;14(1):e0211425. doi: 10.1371/journal.pone.0211425 (PMC6349328; doi:10.1371/journal.pone.0211425)
Supplement: S1 Table — For each day of insulin infusion, the mean number of BG measurements/patient with standard deviation are given. (DOCX) [file pone.0211425.s001.docx]

Table S1: Mean number of blood glucose (BG) measurement per day: comparison between « before or static » and « after or dynamic» periods. For each day of insulin infusion, the mean number of BG measurements/patient with standard deviation are given.

|  | **STATIC**  **mean±SD** | **DYNAMIC mean±SD** | ***p*** |
| --- | --- | --- | --- |
| **D1** | 3.7±1.9 | 4.3±2.3 | 0.07 |
| **D2** | 7.2±1.9 | 7.9±2.7 | 0.09 |
| **D3** | 5.9±2.0 | 7.7±2.1 | **<0.01** |
| **D4** | 6.2±1.9 | 7.0±2.8 | 0.27 |
| **D5** | 6.1±2.4 | 5.8±2.8 | 0.75 |
| **D6** | 6.0±2.9 | 7.2±2.3 | 0.40 |
| **D7** | 6.6±1.7 | 7.2±2.8 | 0.67 |
| **Total** | 5.54±1.1 | 6.01±1.65 | 0.6 |
