# Supplementary material for: Prospective evaluation of a dynamic insulin infusion algorithm for non critically-ill diabetic patients: A before-after study
Source: PLoS One. 2019 Jan 28;14(1):e0211425. doi: 10.1371/journal.pone.0211425 (PMC6349328; doi:10.1371/journal.pone.0211425)
Supplement: S2 Table — For each timing of insulin infusion, the mean BG (mg/dl) with the standard deviation are given. (DOCX) [file pone.0211425.s002.docx]

Table S2: Evolution of mean blood glucose (BG) levels during both before and after periods. For each timing of insulin infusion, the mean BG (mg/dl) with the standard deviation are given.

|  | **Static** | **Dynamic** |
| --- | --- | --- |
| **Overall** |  |  |
| H0 | 247 ± 99 | 258 ± 113 |
| H6 | 192 ± 74 | 175 ± 71 |
| H12 | 168 ± 70 | 166 ± 57 |
| H24 | 203 ± 88 | 204 ± 62 |
| H48 | 191 ± 77 | 217 ± 76 |
| H72 | 188 ± 77 | 187 ± 72 |
| **Vigorous patients** |  |  |
| H0 | 244 ± 101 | 245 ± 110 |
| H6 | 188 ± 062 | 166 ± 076 |
| H12 | 173 ± 77 | 151 ± 47 |
| H24 | 205 ± 65 | 202 ± 75 |
| H48 | 191 ± 82 | 211 ± 54 |
| H72 | 220 ± 67 | 151 ± 63 |
| **Frail patients** |  |  |
| H0 | 249 ± 99 | 269 ± 116 |
| H6 | 194 ± 82 | 179 ± 69 |
| H12 | 164 ± 66 | 176 ± 63 |
| H24 | 202 ± 99 | 205 ± 55 |
| H48 | 191 ± 77 | 219 ± 83 |
| H72 | 176 ± 79 | 203 ± 72 |
