# Supplementary material for: Prospective evaluation of a dynamic insulin infusion algorithm for non critically-ill diabetic patients: A before-after study
Source: PLoS One. 2019 Jan 28;14(1):e0211425. doi: 10.1371/journal.pone.0211425 (PMC6349328; doi:10.1371/journal.pone.0211425)
Supplement: S1 Fig — (PDF) [file pone.0211425.s003.pdf]

Insulin infusion protocol-Department of endocrinology and diabetes-University Hospital Clermont-Ferrand

- Edited in january, 1st, 2010, validity…
- Insulin Aspart 50 UI in 49.50 ml of 0.9% NaCl
- BG or Glycemia: 100 mg/dl=5.5 mmol/l

**Preprandial-nocturnal period**		
**BG (mg/dl)**	**Insulin infusion rate per hour (UI/Hr)**	**Comments**
**<50**	0.1	Consider Hypoglycemia if signs and symptomes<br>Give sugar (15 gr or equivalent: fruit juice) till normalisation of BG<br>IV: 50 ml of G30% over 5 minutes<br>BG measurement every 15-30 minutes
**50-70**	0.3	BG measurement every hour
**71-90**	0.8	BG measurement every 4 hours
**91-120**	1.1	BG measurement every 4 hours
**121-160**	1.4	BG measurement every 4 hours
**161-200**	1.8	BG measurement every 4 hours
**201-250**	2.3	BG measurement every 4 hours
**251-300**	2.7	BG measurement every 2 hours
**301-500**	4	BG measurement every hour<br>Look for ketone bodies (blood or urine)<br>Check IV line permeability
**>500**	6	BG measurement every hour<br>Look for ketone bodies (blood or urine)<br>Check IV line permeability
**During meal**		
Multiply by 1.5 the calculated rate (table above) for 2 hours after meal. Example: if BG 210 mg/dl just before meal, the rate is 2.3X1.5=3.45 UI/Hr for 2 hours.		
If hypoglycemia, give sugar (see comments above) and reduce the rate to 0.1 UI/Hr till normalisation of BG.		
If an adjustment is necessary, according to patient’s status, please call the physician or the resident		

Figure S1: The static insulin infusion protocol used during the “before” period of the study.
