# Supplementary material for: Prospective evaluation of a dynamic insulin infusion algorithm for non critically-ill diabetic patients: A before-after study
Source: PLoS One. 2019 Jan 28;14(1):e0211425. doi: 10.1371/journal.pone.0211425 (PMC6349328; doi:10.1371/journal.pone.0211425)
Supplement: S2 Fig — (PDF) [file pone.0211425.s004.pdf]

Rate of insulin infusion at initiation:

Initial BG (mg/dL) mmol/l	100-140 5.5-7.7	141-180 7.8-10	181-220 10.1-12.2	221 – 260 12.3-14.5	261–300 14.6-16.6	301–350 16.7-19.4	351–400 19.5-22.2	> 400 (« HI » on glycemia reading system) >22.2
Initiation rate (V: IU/h)	1.2	1.5	2	2.5	3 KETONE test	3.5 KETONE test	4 KETONE test	6 KETONE test

Monitor BG one hour after initiation of insulin infusion and adjust the rate (V) according to the table bellow

ELDIIP protocol

BG monitoring rhythme:

-After 1 hour, when starting insulin infusion or if significant modification of the current rate.
-Every 2 hours, with BG before each meal and 2 hours after the meal.
-Every 4 hours is acceptable only after BG stabilization within the target glycemia, on several measures.
-Intensive BG monitoring : if hypoglycemia (cf HYPOGLYCEMIA protocol).

Previous Blood Glucose (BG) value (mg/dl and mmol/l) \ Current Blood Glucose (BG) value (mg/dl and mmol/l)	<70 <3.9	71-100 3.9-5.5	101-140 5.6-7.7 (GLYCEMIC TARGET)	141-180 7.8-10 (GLYCEMIC TARGET)	181-220 10.1-12.2	221-260 12.3-14.5 KETONE test	261-300 14.6-16.6 KETONE test	301-400 16.7-22.2 KETONE test	>400 >22.2 KETONE test
70-100 3.9-5.5	HYPOGLYCEMIA protocol	V÷1.5	No change	No change	V+0.5 IU/h	V+1 IU/h	V+2 IU/h	V+2.5 IU/h	V+3 IU/h
101-140 5.6-7.7		V÷1.5	No change	No change	V+0.5 IU/h	V+1 IU/h	V+1.5 IU/h	V+2 IU/h	V+3 IU/h
141-180 7.8-10		V÷1.5	V-0.5 IU/h	No change	V+0.5 IU/h	V+0.5 IU/h	V+1 IU/h	V+1.5 IU/h	V+2.5 IU/h
181-220 10.1-12.2		V÷2	V-0.5 IU/h	No change	No change	V+0.5 IU/h	V+1 IU/h	V+1.5 IU/h	V+2 IU/h
221-260 12.3-14.5		V÷2	V-1 IU/h	V-0.5 IU/h	No change	V+0.5 IU/h	V+1 IU/h	V+1.5 IU/h	V+2 IU/h
261-300 14.6-16.6		V÷3	V-2 IU/h	V-1 IU/h	V-0.5 IU/h	No change	V+1 IU/h	V+2 IU/h	V+3 IU/h
301-400 16.7-22.2		V÷3	V-2 IU/h	V-1.5 IU/h	V-1.5 IU/h	V-1 IU/h	V+1 IU/h	V+2 IU/h	V+3 IU/h
>400 >22.2		V÷4	V-3 IU/h	V-3 IU/h	V-2.5 IU/h	V-2 IU/h	No change	No change	V+3 IU/h

HYPOGLYCEMIA protocol
If BG <70 mg/dl :
1-Note the insulin infusion rate (V/h) before hypoglycemia.
2-Decrease the rate to 0.1 IU/h.
3-Give sugar immediately:
-20g if BG≤50 mg/dl.
-10 g if BG 50<BG≤70 mg/dl.
4-Check BG after 30 minutes until the glycemia being above 100mg/dl. Give (10g) sugar again if glycemia remain under 100mg/dl.
5-Restart insulin infusion at rate =V/h÷2 and apply the algorithm to determine insulin infusion rate.

Instead of SUGAR
-Replace 20g of sugar by 60 mL of G30% or 180 ml of G10% if needed.
-Replace 10g of sugar=30 mL of G30 or 90 ml of G10% if needed.

VIGOROUS PATIENT

VIGOROUS PATIENT

*** To prepare insulin infusion for electrical pump, use Aspart, Lispro or Glulisine in a syringe of 50ml to prepare a concentration of 1IU/mL.**
*** Current insulin rate:** the calculation of each case give the insulin infusion rate to apply.
*** If calculated insulin rate=0 IU/h:** continue infusion at 0.1 IU/h.
***For MEDICAL ALARM cf page 2**

EQUIVALENCE BETWEEN BLOOD KETONE AND URINE KETONE RATE:

**Blood Ketone rate in mmol/l :**	≤ 0.3	0.4 – 0.6	0.6 – 1.5	1.5 – 3	>3
**Urine Ketone rate:**	0	+	++	+++	++++

Figure S2: Frontpage of the dynamic algorithm for insulin infusion adapted for vigorous patients.
