# Supplementary material for: Prospective evaluation of a dynamic insulin infusion algorithm for non critically-ill diabetic patients: A before-after study
Source: PLoS One. 2019 Jan 28;14(1):e0211425. doi: 10.1371/journal.pone.0211425 (PMC6349328; doi:10.1371/journal.pone.0211425)
Supplement: S3 Fig — (PDF) [file pone.0211425.s005.pdf]

Rate of insulin infusion at initiation:

Initial BG (mg/dL)<br>mmol/l	100-140<br>5.5-7.7	141-180<br>7.8-10	181–220<br>10.1-12.2	221–260<br>12.3-14.5	261–300<br>14.6-16.6	301–350<br>16.7-19.4	351–400<br>19.5-22.2	> 400 (« HI » on glycemia reading system)<br>> 22.2
Initiation rate<br>(V: IU/h)	1.0	1.4	1.8	2.2	2.6<br>KETONE test	3.0<br>KETONE test	3.5<br>KETONE test	4<br>KETONE test

Monitor BG one hour after initiation of insulin infusion and adjust the rate (V) according to the table bellow

ELDIIP protocol

Current Blood Glucose (BG) value (mg/dl and mmol/l)

BG monitoring rhythme: -After 1 hour, when starting insulin infusion or if significant modification of the current rate.
-Every 2 hours, with BG before each meal and 2 hours after the meal.
-Every 4 hours is acceptable only after BG stabilization within the target glycemia, on several measures.
-Intensive BG monitoring: if hypoglycemia (cf HYPOGLYCEMIA protocol).

Previous Blood Glucose (BG) value (mg/dl and mmol/l) \ Current BG	<100<br><5.5	101-140<br>5.5-7.7	**141-180<br>7.8-10**<br>GLYCEMIC TARGET	**181-220<br>10.1-12.2**<br>GLYCEMIC TARGET	221-260<br>12.3-14.5	261-300<br>14.6-16.6<br>KETONE test	301-400<br>16.7-19.4<br>KETONE test	> 400<br>> 22.2<br>KETONE test
101-140<br>5.6-7.7		V÷1.5	No change	V+0.5 IU/h	V+1 IU/h	V+1.5 IU/h	V+2 IU/h	V+2.5 IU/h
**141-180<br>7.8-10**		V÷2	No change	V+0.5 IU/h	V+1 IU/h	V+1.5 IU/h	V+1.5 IU/h	V+2 IU/h
**181-220<br>10.1-12.2**		V÷2.5	V-0.5 IU/h	No change	V+0.5 IU/h	V+1 IU/h	V+1.5 IU/h	V+2 IU/h
221-260<br>12.3-14.5		V÷2.5	V-1.5 IU/h	No change	V+0.5 IU/h	V+1 IU/h	V+1.5 IU/h	V+2 IU/h
261-300<br>14.6-16.6		V÷3	V-2 IU/h	V-1 IU/h	V-0.5 IU/h	V+0.5 IU/h	V+1 IU/h	V+2 IU/h
301-400<br>16.7-22.2		V÷4	V-2.5 IU/h	V-2 IU/h	V-1 IU/h	V+0.5 IU/h	V+1 IU/h	V+2 IU/h
> 400<br>> 22.2		V÷4	V-3 IU/h	V-2.5 IU/h	V-2 IU/h	No change	No change	V+2.5 IU/h

HYPOGLYCEMIA protocol

If BG <100 mg/dl:

1-Note the insulin infusion rate (V/h) before hypoglycemia.

2-Decrease the rate to 0.1 IU/h.

3-Give sugar immediately:
  -20g if BG≤70 mg/dl.
  -10 g if BG 70<BG≤100 mg/dl.

4-Check BG after 30 minutes until the glycemia being above 100mg/dl. Give (10g) sugar again if glycemia remain under 100mg/dl.

5-Restart insulin infusion at rate =V/h÷2 and apply the algorithm to determine insulin infusion rate.

Instead of SUGAR

-Replace 20g of sugar by 60 mL of G30% or 180 ml of G10% if needed.

-Replace 10g of sugar=30 mL of G30 or 90 ml of G10% if needed.

FRAIL PATIENT

FRAIL PATIENT

*** To prepare insulin infusion for electrical pump, use Aspart, Lispro or Glulisine in a syringe of 50ml to prepare a concentration of 1IU/mL.**

*** Current insulin rate:** the calculation of each case give the insulin infusion rate to apply.

*** If calculated insulin rate=0 IU/h:** continue infusion at 0.1 IU/h.

***For MEDICAL ALARM cf page 2**

EQUIVALENCE BETWEEN BLOOD KETONE AND URINE KETONE RATE:

**Blood Ketone rate in mmol/l :**	≤ 0.3	0.4 – 0.6	0.6 – 1.5	1.5 – 3	>3
**Urine Ketone rate:**	0	+	++	+++	++++

Figure S3: Frontpage of the dynamic algorithm for insulin infusion adapted for frail patients.
