# Supplementary material for: Prospective evaluation of a dynamic insulin infusion algorithm for non critically-ill diabetic patients: A before-after study
Source: PLoS One. 2019 Jan 28;14(1):e0211425. doi: 10.1371/journal.pone.0211425 (PMC6349328; doi:10.1371/journal.pone.0211425)
Supplement: S4 Fig — (PDF) [file pone.0211425.s006.pdf]

FRAIL PATIENT/VIGOROUS PATIENT

SITUATIONS REQUIRING PHYSICIAN'S INTERVENTION:

1-Hyperglycemia (>250 mg/dl or 13.9 mmol/l) with ketone bodies in blood (>1.2mmol/L) or urine (≥ ++); 2-Persistent hyperglycemia> 400mg/dl (22 mmol/l) despite 2 adjustments of insulin rate; 3-Persistent hyperglycemia despite insulin infusion above >8 IU/h in a frail patient *(>10UI/h for vigorous patient)*; 4-Persistent hypoglycemia despite two successive sugar supplimentations; 5- when a personnalized ajustment is required (rate of bolus SC, infusion rate or BG monitoring...).

SUB-CUTANEOUS BOLUS BEFORE MEAL:

Insulin bolus is delivered only if the patient takes his meal and not in hypoglycemia, it must be READJUSTED DAILY after medical advice if the post-prandial glycemia is out of target.

Use for this sub cutaneous insulin injection either ASPART, LISPRO or GLULISINE.

Table of corresponding bolus doses according to the weight (0.07UI/kg):

**Weight kg**	**40**	**45**	**50**	**55**	**60**	**65**	**70**	**75**	**80**	**85**	**90**	**95**	**100**	**105**	**110**	**115**	**120**
**Units of fast insulin analog for SC bolus before meal**	**3**	**3**	**4**	**4**	**4**	**5**	**5**	**5**	**6**	**6**	**6**	**7**	**7**	**7**	**8**	**8**	**8**

SIGNIFICANT VARIATION OF INSULIN INFUSION RATE :

V= current insulin infusion rate

New insulin infusion rate (new V)

If the new insulin infusion rate to apply is below or equal (≤) to the rate figuring on the left column with the current insulin infusion rate, or if the new insulin infusion rate to apply is above or equal (≥) to the rate figuring on the right column, the variation of insulin rate is considered as significant and it is suggested to check BG in the following hour.

Example :

1/ V= 1.4 IU/h and I have to apply a new insulin infusion rate ≤0.6, the decrease of insulin rate variation is significant.

2/ V= 1.4 and I have to apply a new insulin infusion rate between 0.6 and 2.2, the variation of insulin rate is so not significant.

3/ V= 1.4 and I have to apply a new rate insulin infusion ≥2.2, the increase of insulin rate variation is significant.

MEDICAL ALARM if V > 8UI/h for frail patient or >10UI/h for vigorous patient.

New V ≤	V	New V ≥	New V ≤	V	New V ≥	New V ≤	V	New V ≥	New V ≤	V	New V ≥	New V ≤	V	New V ≥	New V ≤	V	New V ≥
0.2	**0.5**	0.8	0.9	**2.1**	3.3	1.9	**3.7**	5.6	3.4	**5.3**	7.2	4.8	**6.9**	9.0	6.4	**8.5**	10.6
0.2	**0.6**	1.0	1.0	**2.2**	3.4	1.9	**3.8**	5.7	3.5	**5.4**	7.3	4.9	**7.0**	9.1	6.5	**8.6**	10.8
0.3	**0.7**	1.1	1.0	**2.3**	3.6	2.0	**3.9**	5.8	3.6	**5.5**	7.4	5.0	**7.1**	9.2	6.5	**8.7**	10.9
0.3	**0.8**	1.3	1.1	**2.4**	3.7	2.1	**4.0**	5.9	3.6	**5.6**	7.6	5.0	**7.2**	9.4	6.6	**8.8**	11.0
0.4	**0.9**	1.4	1.1	**2.5**	3.9	2.2	**4.1**	6.0	3.7	**5.7**	7.7	5.1	**7.3**	9.5	6.7	**8.9**	11.1
0.4	**1.0**	1.6	1.2	**2.6**	4.0	2.3	**4.2**	6.1	3.8	**5.8**	7.8	5.2	**7.4**	9.6	6.8	**9.0**	11.3
0.4	**1.1**	1.8	1.2	**2.7**	4.2	2.4	**4.3**	6.2	3.8	**5.9**	8.0	5.3	**7.5**	9.6	6.8	**9.1**	11.4
0.5	**1.2**	1.9	1.3	**2.8**	4.3	2.4	**4.4**	6.4	3.9	**6.0**	8.1	5.3	**7.6**	9.7	6.9	**9.2**	11.5
0.5	**1.3**	2.1	1.3	**2.9**	4.5	2.5	**4.5**	6.5	4.0	**6.1**	8.2	5.8	**7.7**	9.7	7.0	**9.3**	11.6
0.6	**1.4**	2.2	1.4	**3.0**	4.7	2.5	**4.6**	6.6	4.0	**6.2**	8.4	5.9	**7.8**	9.8	7.1	**9.4**	11.8
0.6	**1.5**	2.4	1.6	**3.1**	4.7	2.8	**4.7**	6.6	4.1	**6.3**	8.5	5.9	**7.9**	9.9	7.1	**9.5**	11.9
0.6	**1.6**	2.6	1.6	**3.2**	4.8	2.9	**4.8**	6.7	4.2	**6.4**	8.5	6.0	**8.0**	10.0	7.2	**9.6**	12.0
0.7	**1.7**	2.7	1.7	**3.3**	5.0	2.9	**4.9**	6.9	4.2	**6.5**	8.6	6.1	**8.1**	10.1	7.3	**9.7**	12.1
0.7	**1.8**	2.9	1.7	**3.4**	5.1	3.0	**5.0**	7.0	4.4	**6.6**	8.6	6.2	**8.2**	10.3	7.4	**9.8**	12.3
0.8	**1.9**	3.0	1.8	**3.5**	5.3	3.1	**5.1**	7.1	4.7	**6.7**	8.7	6.2	**8.3**	10.4	7.4	**9.9**	12.4
0.8	**2.0**	3.2	1.8	**3.6**	5.4	3.4	**5.2**	7.1	4.8	**6.8**	8.8	6.3	**8.4**	10.5	7.5	**10.0**	12.5

The algorithm (paper version) is available in the quality documents of your unit.

In case of difficulty, contact the departments of diabetes or pharmacy

Figure S4: Backpage of the algorithm with specific protocol recommendations.
